# ﻿*Plagiotheciumtalbotii*, a new species from the Aleutian Islands (Alaska, U.S.A.)

**DOI:** 10.3897/phytokeys.194.81652

**Published:** 2022-04-15

**Authors:** Grzegorz J. Wolski, Paulina Nowicka-Krawczyk, William R. Buck

**Affiliations:** 1 Department of Geobotany and Plant Ecology, Faculty of Biology and Environmental Protection, University of Lodz, ul. Banacha 12/16, 90-237 Lodz, Poland; 2 Department of Algology and Mycology, Faculty of Biology and Environmental Protection, University of Lodz, ul. Banacha 12/16, 90-237 Lodz, Poland; 3 Institute of Systematic Botany, The New York Botanical Garden, Bronx, NY 10458-5126, USA

**Keywords:** Attu Island, Bryophyta, Plagiotheciaceae, S. S. Talbot, taxonomy

## Abstract

*Plagiotheciumtalbotii***sp. nov.** is described from Attu Island, Alaska, U.S.A. The newly-described species is not similar in appearance to any Northern Hemisphere species; only the habit is similar to *P.platyphyllum*. However, it not only occupies a different habitat than that species, but genetically and morphologically, it is clearly distinct from it. The results of DNA sequencing, a detailed description of the morphological features, illustrations, ecological preferences and currently known geographical distribution of *P.talbotii* are presented. The most important distinguishing morphological features of this species are: the size of the plant; dimensions and symmetry of the leaves; dimensions of cells and their areolation; entire leaf apex; and long decurrencies with some inflated cells. Additionally, we propose to place *P.talbotii* in section Plagiothecium, which is confirmed by genetic analysis and morphological features.

## ﻿Introduction

Herbarium collections are unquestionably a rich and very important source of data on the ecology and distribution of plants. Revisions based on herbarium material are the primary source for taxonomic research. Now, having a number of methods at our disposal (e.g. genetic analyses, mathematical modelling, SEM), integrative taxonomy sheds new light not only on the status or distribution of the taxa studied, but also on the relationships between them and not only at the species level (e.g. Huttunen et al. 2018; Guerra et al. 2019; Ignatov et al. 2020; Plášek and Ochyra 2020; Vigalondo et al. 2020; Wolski and Nowicka-Krawczyk 2020; Wolski et al. 2020; Melamed et al. 2021).

Despite the fact that the Northern Hemisphere is relatively well studied botanically, there are still many areas that are blank spots for this part of the world. The same is true for bryophytes as for vascular plants, but even more so. Many species, genera and even families require basic research related to their variability, distribution, ecology or taxonomic status (Anderson et al. 1990; Ignatov et al. 2006; Hodgetts 2015; Suzuki 2016; Hodgetts and Lockhart 2020; Wolski et al. 2021a, b).

The genus *Plagiothecium* Schimp. which currently has been divided by Wynns et al. (2018) into nine sections (*Leptophyllum* Jedl.; *Lycambium* Jedl.; *Ortholimnobium* (Dixon) J.T. Wynns; *Orthophyllum* Jedl.; *Plagiothecium*; *Pseudo*-*Neckera* (Kindb.) J.T. Wynns; *Rectithecium* (Hedenäs & Huttunen) J.T. Wynns; *Saviczia* (Abramova & I.I. Abramov) Z. Iwats.; *Struckia* (Müll. Hal.) J.T. Wynns) over the past decades may give the impression of being well-researched. However, the genus was described as fairly species-poor and represented in the Northern Hemisphere by only a dozen taxa (e.g. Ireland 1969, 1986; Iwatsuki 1970; Lewinsky 1974; Smith 2001). The understanding of *Plagiothecium* has changed relatively recently, when DNA-based research proved that many of the taxa have been too broadly circumscribed (Zuo et al. 2011; Wynns et al. 2018; Ignatova et al. 2019; Wolski and Nowicka-Krawczyk 2020) and that the bryoflora of North America, Europe and Asia is much richer in *Plagiothecium* than previously assumed (e.g. Wynns et al. 2018; Ignatova et al. 2019; Wolski, Jukoninė 2019; Wolski 2020a, b; Müller and Wynns 2020; Wolski and Nowicka-Krawczyk 2020; Wolski et al. 2021b).

However, despite extensive research now being carried out, the number of Northern Hemisphere species still seems to be underestimated. Alaska, including the Aleutian Islands, are a “blank spot” in our knowledge of mosses, including the genus *Plagiothecium*. As a result of the taxonomic revision of *Plagiothecium* from the Aleutians, we recently described a new species, *Plagiotheciumschofieldii* (Wolski et al. 2021a). Examination of additional material resulted in yet another undescribed species being discovered.

## ﻿Materials and methods

### ﻿Taxonomic analyses

Specimens from the Missouri Botanical Garden (MO), the University of British Columbia (UBC) and The New York Botanical Garden (NY) were analysed during the revision of *Plagiothecium* from the Aleutian Islands. A single specimen (MO*5925637*) was selected for DNA analysis, with the appropriate consent from the herbarium curator.

### ﻿DNA isolation, amplification and sequencing

Total DNA was extracted using the GeneMATRIX Plant & Fungi DNA Purification Kit (Eurx, Gdansk, Poland) following the manufacturer’s protocol. Three equal samples of 20 mg of dry tissue from the leafy stems of bryophytes were homogenised in the lysis buffer, delivered by the purification kit, using a hand-held stainless steel homogeniser (Schlüter Biologie, Eutin, Germany). DNA extracts were quantified with a BioDrop DUO Spectrophotometer (BioDrop Ltd, Cambridge, U.K.) and the sample with high quality DNA (1.7–1.9 OD_260_/OD_280_) was selected for further analysis.

The molecular research was based on nuclear and chloroplast DNA markers: ITS (from the 3´ end of the nuclear spacer ITS1, through the 5.8S rDNA, to the 5´ end of the ITS2 spacer); and *rpl16* cpDNA gene encoding ribosomal protein L16. Markers were selected, based on Wynns et al. (2018), Wolski and Nowicka-Krawczyk (2020) and Wolski et al. (2021) from *Plagiothecium*-focused studies.

All markers were amplified by PCR in a few replicates to obtain high quality amplicons for sequencing. PCR was performed using primers and reaction conditions as described in Wolski et al. (2021).

PCR products were visualised on an agarose gel (1.5%, 90V, 40 minutes) stained with GelRED fluorescent dye (Biotum, Fremont, CA, U.S.A.) and two replicates of each marker per sample were chosen for sequencing. Amplicons from the PCR reaction were cleaned using Syngen Gel/PCR Mini Kit (Syngen Biotech, Wrocław, Poland) according to the manufacturer’s protocol. Samples were sequenced with Sanger sequencing, using primers from amplification by SEQme s.r.o. company (Dobris, Czech Republic). The obtained sequences were assembled in Geneious 11.1.5 (Biomatters Aps, Aarhus, Denmark) (http://www.geneious.com). The sequences were submitted to the NCBI GenBank database (www.ncbi.nlm.nih.gov) under the accession numbers OM337522 for ITS and OM311940 for *rpl*16.

### ﻿Phylogenetic analyses

Phylogenetic analyses of the studied specimen and other species in the *Plagiothecium* group (Table 1) were performed, based on a concatenated ITS-*rpl16* sequence matrix. Sequences were aligned using the MAFFT v. 7 web server (Katoh et al. 2017) (http://mafft.cbrc.jp/alignment/server/) where the auto strategy was applied, the scoring matrix of 200PAM with Gap opening penalty of 1.53, UniREf50 for Maft-homologs and Plot and alignment with threshold of 39 score were set. The obtained alignments were checked for poorly and ambiguously aligned regions and small corrections were made by eye. The evolutionary models were calculated using PartitionFinder 2 software (Lanfear et al. 2016), chosen according to the Akaike Information Criterion (Table 2).

**Table 1.** Voucher information and accession numbers for the specimens included in the phylogenetic analyses.

Taxon	Collection	Locality	ITS	rpl16
*Isopterygiopsispulchella*	UC barcode 1947397	USA: CA	KY550336	KY514042
*P.angusticellum*	*Wolski 5*	Poland	MN077501	MN311136
*P.angusticellum*	*Wolski 22*	Poland	MN077507	MN311142
*P.angusticellum*	*Wolski 23*	Poland	MN077508	MN311143
*P.angusticellum*	*Wolski 25*	Poland	MN077510	MN311145
*P.angusticellum*	*Wolski 26*	Poland	MN077511	MN311146
*P.angusticellum*	*Wolski 29*	Poland	MN077513	MN311148
*P.brasiliense*	E barcode 00387968	Brazil	KY550266	KY513971
*P.cavifolium*	CP:*J.T. Wynns 1885*	Denmark: Sjaelland	KF882225	KF882325
*P.cavifolium*	CP:*J.T. Wynns 2960*	Germany: Schauinsland, Hochschwarzwald	KF882226	KF882326
*P.conostegium*	NY:*S.P. Churchill et al. 19839*	Bolivia	KY550271	KY513976
*P.conostegium*	NY barcode 00845279	Guatemala	KY550318	KY514024
*P.conostegium*	S-B53327	Mexico	KY550272	KY513977
*P.denticulatum*	CP:*J.T. Wynns 2081*	Denmark: Sjælland, Sorø Kommune	KF882229	KF882329
*P.denticulatum*	BONN:*O.M. Afoninas.n.*	Russia: Far East, Chukotka	KY550275	KY513980
*P.denticulatum*	C:*R.R. Ireland 23098*	Canada: ON	KY550276	KY513981
P.denticulatumvar.bullulae	UC barcode 1798690	USA: NV	KY550278	KY513983
P.denticulatumvar.bullulae	UC barcode 1947417	USA: CA	KY550277	KY513982
P.denticulatumvar.obtusifolium	CP:*J.T. Wynns 2842*	Germany: Hochschwarzwald, Schauinsland	KF882230	KF882330
P.denticulatumvar.obtusifolium	UC barcode 1724036	USA: WA	KY550279	KY513984
P.denticulatumfo.pungens	DUKE barcode 0150010	USA: Alaska, Simenof Island	KY550280	KY513985
*P.japonicum*	DUKE barcode 0172241	USA: Alaska, Simenof Island	KY550291	KY513996
*P.lamprostachys*	S-B54613	Australia: VIC	KY550284	KY513989
*P.lamprostachys*	DUKE barcode 0156846	Australia: VIC	KY550285	KY513990
*P.lamprostachys*	S:*H. Streimann 47719*	Australia: NSW	KY550282	KY513987
*P.longisetum*	*Wolski 12*	Poland	MN077502	MN311137
*P.longisetum*	*Wolski 14*	Poland	MN077503	MN311138
*P.longisetum*	*Wolski 15*	Poland	MN077504	MN311139
*P.longisetum*	*Wolski 19*	Poland	MN077506	MN311141
*P.membranosulum*	BONN:*J.*-*P. Frahm7756*	Democratic Republic of the Congo	KY550310	KY514015
*P.membranosulum*	S-B78514	South Africa	KY550303	KY514008
*P.membranosulum*	DUKE barcode 0016754	South Africa	KY550304	KY514009
*P.nemorale*	CP:*J.T. Wynns 3044*	Germany: Farnberg, Schwarzwald-Baar	KF882239	KF882339
*P.nemorale*	CP: *J.T. Wynns 2684*	Germany: Mooswald, Kaiserstuhl	KF882237	KF882337
*P.nemorale*	*Mishler 3835*	Iran: Sisangan National Park, Manzandaran Prov.	KF882238	KF882338
*P.ovalifolium*	DUKE barcode 0188886	Chile	KY550314	KY514019
*P.platyphyllum*	C:*J. Lewinsky et al. s.n.*	Finland: Haluna, Nilsiae, Savonia borealis	KF882241	KF882341
*P.ruthei*	CP: *J.T. Wynns 1997*	Denmark: Sj*æ*lland, Lyngby Aamose	KF882242	KF882342
*P.talbotii*	*W.B. Schofield*, *S.S. Talbot 120206*, MO*5925637* (dupl. UBC		OM337522	OM311940

Phylogenetic calculations were performed using Maximum Likelihood analysis (ML) in the IQ-TREE web server (Trifinopoulos et al. 2016) (http://iqtree.cibiv.univie.ac.at/) with the ultrafast bootstrap (UFBoot) pseudolikelihood algorithm (Hoang et al. 2018) and 10000 replicates; and Bayesian Inference (BI) in MrBayes 3.2.2 (Ronquist et al. 2012) where two parallel Markov Chain Monte Carlo (MCMC) runs for four million generations each, with trees sampled every 1000 generations. The average standard deviation of split frequencies in both cases remained below 0.01 for the last 1000 generations and posterior probabilities were estimated from the 50% majority-rule consensus tree after elimination of the first 25% of samples as burn-in. The alignment and tree files were submitted to the figshare online database (https://doi.org/10.6084/m9.figshare.18586082.v1).

**Table 2.** Summary of partitions for ITS-*rpl*16 matrix (1526 bp) evolutionary model selection and phylogenetic interference using PartitionFinder2.

	ITS1	5.8S gDNA	ITS2	*rpl*16 intron	*rpl*16 codon
ML	F81	JC	HKY +I	TN+I	F81
BI	F81	JC	HKY	GTR	F81

Haplotype network analysis was performed using Median Joining Network in PopART v. 1.7 with gap coding as a single event, irrespective of length and haplotype geographic distribution (Leigh and Bryant 2015).

## ﻿Results and discussion

**Figure 1.**
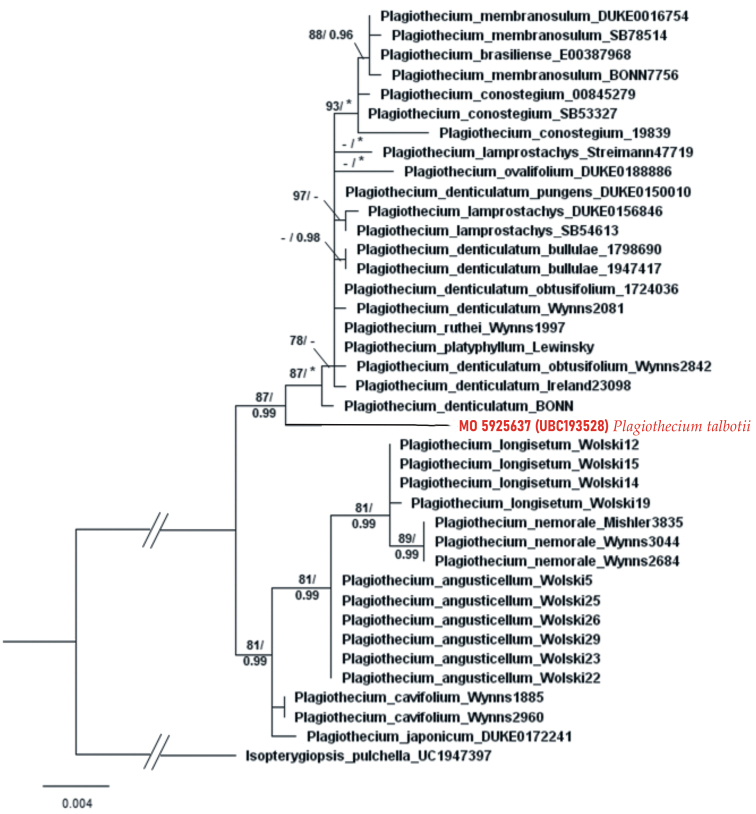
Phylogenetic tree of Plagiothecium taxa (sect. Plagiothecium [upper] and *Orthophyllum* [lower clade] with *Isopterygiopsispulchella* as the outgroup, based on concatenated nuclear (ITS1-5.8S-ITS2) and chloroplast (*rpl*16) DNA markers (total 1526 bp). The tree presents the position of the studied specimen from Alaska amongst *Plagiothecium*. Numbers on branches indicate bootstrap values from ML followed by posterior probabilities from BI analysis. An asterisk (*) indicates 100 (ML) and 1.00 (BI), while a minus sign (-) indicates values below 75 (ML) and 0.95 (BI). The topology of the tree was based on ML analysis.

Phylogenetic analyses using the concatenated ITS-*rpl*16 matrix placed this Alaskan specimen within the sister branch of the sect. Plagiothecium clade with high support from Bayesian Inference (PP = 0.99), but slightly lower support from Maximum Likelihood (BS = 87) (Fig. 1). Although the topology of the tree shows clear distinction of the examined material, the closest representative with maximum support from BI to *P.talbotii* is the specimen BONN: *O.M*. *Afonina s.n*. described as *P.denticulatum*.

**Figure 2.**
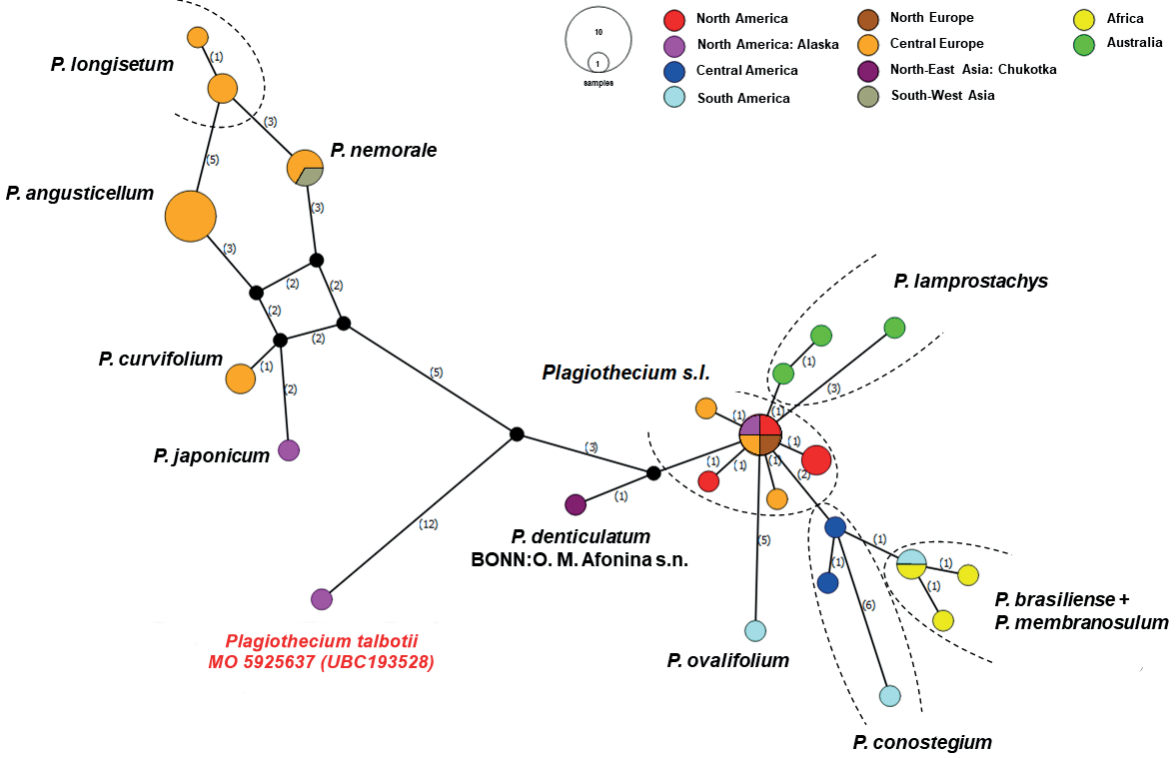
Median-joining haplotype network of *Plagiothecium* [bottom-right] and *Orthophyllum* sections [top-left] of *Plagiothecium* constructed in PopART. Haplotypes are represented by circles with colours indicating geographic distribution. Numbers on branches indicate the mutational steps.

The haplotype network (Fig. 2) also confirms a closer relationship of *P.talbotii* with sect. Plagiothecium than with representatives from sect. Orthophyllum; however, the position of *P.talbotii* is isolated. The lowest number of mutational steps to *P.talbotii* as mentioned earlier is the specimen BONN: *O.M*. *Afonina s.n*. of *P.denticulatum*. This material was collected from the Far East of Russia – from Chukotka (Table 1), a geographic region adjacent to Alaska.

Although DNA analysis places the tested specimen as a sister clade to the clade represented by taxa from section Plagiothecium, it is morphologically different from most of the representatives of this section.

The shape of the decurrent angular cells is one of the most important features that divide the taxa of this genus into individual sections (Wynns et al. 2018; Wolski et al. 2021a, b). Almost all taxa from sect. Plagiothecium form distinct auricles, but not *P.talbotii*. The alar regions in this species are long and composed of sometimes inflated cells, a feature common to both *P.talbotii* and *P.platyphyllum* (Nyholm 1965; Lewinsky 1974; Smith 2001).

Having leaves shrunken in a dry condition and symmetrical make *P.talbotii* resemble, amongst the Northern Hemisphere of *Plagiothecium*, the previously-mentioned *P.platyphyllum* (Lewinsky 1974; Smith 2001). However, *P.talbotii* is distinguished from *P.platyphyllum* by the size of the leaves; lack of serration near leaf apex; lack of a group of eroded, thin-walled cells (nematocysts) and rhizoids near the leaf apex; dimensions of laminal cells; and habitat occupied by the species (Nyholm 1965; Lewinsky 1974; Smith 2001).

Amongst the Northern Hemisphere species belonging to sect. Plagiothecium at present, there is only one species characterised by a symmetrical leaf: *P.schofieldii*. However, for example, the size and appearance of the turf; the lack of shrinkage and leaf shape, i.e. their concavity and serration and the shape of the decurrent angular cells differentiates this species from *P.talbotii* (Wolski et al. 2021b).

Other species of this section are characterised by asymmetrical to slightly asymmetrical leaves and a completely different set of features when compared with *P.talbotii* (e.g. Lewinsky 1974; Smith 2001; Wolski et al. 2021b).

Taking into account the above facts, we believe that this species belongs to Plagiotheciumsect.Plagiothecium. Thus, we consider that all the above morphological data, supported by molecular studies, warrant the recognition of the Aleutian samples as a new species.

## ﻿Taxonomy

### ﻿ Plagiothecium talbotii

Taxon classificationPlantaeHypnalesPlagiotheciaceae

G.J.Wolski & W.R.Buck sp. nov.

697794DB-54C8-55C1-AB1E-ABD77AE47E0A

#### Type.

U.S.A. Alaska, Attu Island, Lake Elwood area, under tall herbs on slope, 52°51'N, 173°10'E, 14 Sep 2002, *W.B. Schofield* and *S.S. Talbot 120206*, ***holotype***MO*5925637*, ***isotype***UBC B193528.

#### Description.

**Figure 3.**
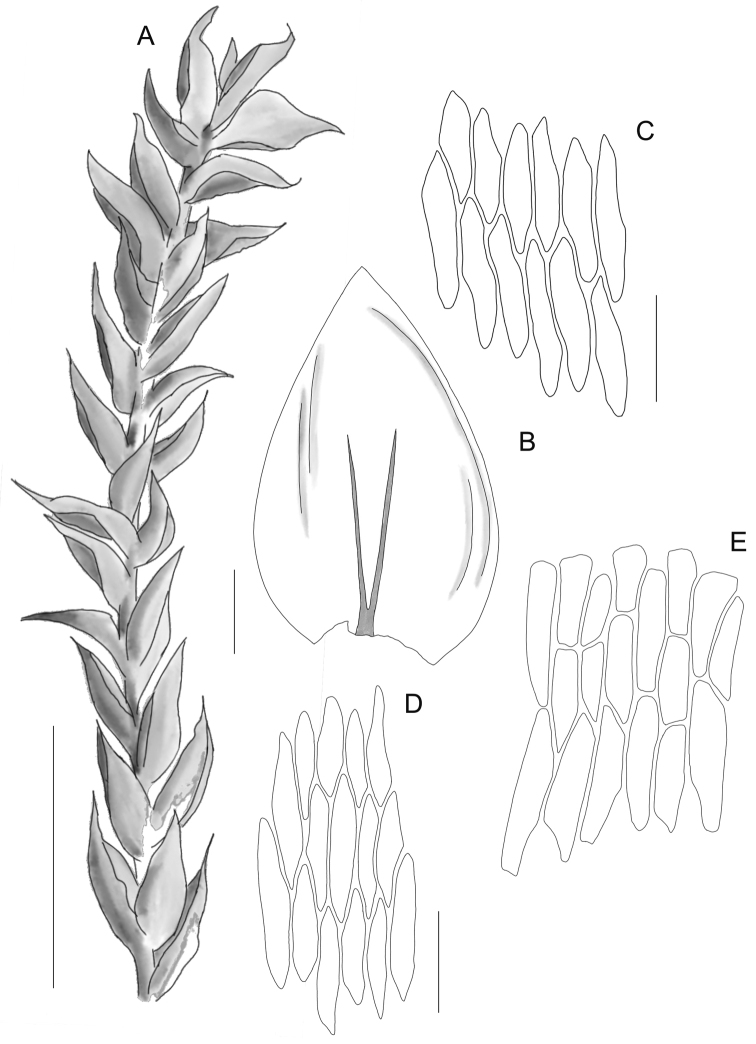
The most important taxonomic features of *Plagiotheciumtalbotii*. Stem (**A**) in dry condition; leaves from the middle of the stem (**B**), dimensions of cells from the apex (**C**), the middle (**D**) and basal part of the leaf (**E**). Drawing by G.J. Wolski from the holotype (*W.B*. *Schofield* and *S.S.Talbot 120206*, MO*5925637* [dupl. UBC B193528]). Scale bars: 1 cm (**A**); 1000 µm (**B**); 100 µm (**C–E**).

Plants large, dark green, without metallic luster, forming loose mats. Stems erect, julaceous in the lower part, more complanate above, 3.0–5.0 cm long (Fig. 3), very thick, in cross-section rounded, with a diameter of 500–700 μm, the central strand very well developed, epidermal cells 16–43 (M 25) × 12–39 (M 25) μm, the parenchyma thin-walled, 25–50 (M 36) × 18–60 (M 37) μm; leaves large, concave, symmetrical, ovate, imbricate, in wet condition, rather closely arranged on the stem, shrunken and sticking out when dry, those leaves from the middle of the stem 3.8–5.0 (M 4.4) mm long and the width measured at the widest point 1.9–3.1 mm (M 2.4); the apex obtuse and apiculate, entire, not denticulate; costae two, thick, strong and very large, extending usually more than ½ of the leaf length, reaching 1.0–3.0 mm (M 2.0); laminal cells rather symmetrical, in unregulated transverse rows, the length and width very variable, but dependent on location: 83–137 (M 101) × 17–22 (M 19) μm at apex, 100–175 (M 139) × 18–32 (M 24) μm at mid-leaf and 88–197 (M 132) × 22–35 (M 28) μm towards insertion, cell areolation loose; decurrencies very long, 700–1000 μm, composed of 3–4 rows of rectangular, at least some gently inflated cells, 90–216 (M 143) × 24–34 (M 28) μm. Sporophytes unknown so far.

#### Etymology.

The present species is named in honour of Stephen S. Talbot who spent decades studying the northern regions of North America, including the Aleutian Islands and who, with Wilfred B. Schofield on 14 September 2002, collected the specimen (No. *120206*), chosen here as the holotype of *Plagiotheciumtalbotii*.

#### Distribution and ecology.

*Plagiotheciumtalbotii* so far has only been recorded from Attu Island in Alaska. In this area, it has been recorded in a non-forested area, on a slope, under tall herbs.

## Supplementary Material

XML Treatment for Plagiothecium talbotii
